# Increasing serum alkaline phosphatase is associated with bone deformity progression for patients with polyostotic fibrous dysplasia

**DOI:** 10.1186/s13018-020-02073-y

**Published:** 2020-12-03

**Authors:** Jun Wang, Zhiye Du, Dasen Li, Rongli Yang, XiaodongTang, Taiqiang Yan, Wei Guo

**Affiliations:** Musculoskeletal Tumor Center, Peking University People’s Hospital, No. 11 Xizhimen South Street, Beijing, 100044 China

**Keywords:** Fibrous dysplasia, Surgery, Pathological fracture, Shepherd’s crook deformity, Bone turnover

## Abstract

**Background:**

Fibrous dysplasia (FD) is a rare bone disorder in which normal intramedullary bone is replaced by fibro-osseous tissue, which is complicated by the progression of Shepherd’s crook deformity. How to predict the progression of Shepherd’s crook deformity is still a challenging for the orthopedic surgeon.

**Methods:**

A total of 159 cases were reviewed in the retrospective study between January 2000 and September 2016. Clinical and monitoring data were collected. We analyzed the correlationship between the bone turnover markers and other parameters (age, gender, FD type, deformity, BMI, and lesion location).

**Results:**

Age, gender, lesion location, lesion type, and shepherd’s crook deformity had a close relationship with preoperative ALP level in the univariate analysis, and the multivariate analysis showed age, gender, lesion type, and shepherd’s crook deformity had the significant relationship with the preoperative serum ALP level. The surgery could remove the bone lesion and suppressed the abnormal bone metabolism. Furthermore, the preoperative ALP level of FD patients with the shepherd’s crook deformity was obviously higher than that without deformity, and the preoperative calcium and phosphorus levels of FD patients with deformity were significantly lower than that without deformity. Notably, for some patients with progression of the shepherd’s crook deformity during the follow-up, ALP increased to the high level and at that time X-ray showed the shepherd’s crook deformity severely progressing.

**Conclusions:**

PFD with higher serum ALP level has obvious tendency to progress severely, and risk factors of progression to the deformity are the condition of bony metabolism and FD type. The deformity of PFD may be related to high speed of bone turnover, which is exactly reflected by the levels of serum ALP and calcium. Evaluation of patients with FD should include a thorough evaluation of calcium/phosphate metabolism and bone turnover.

## Introduction

Fibrous dysplasia of bone (FD) is a nonmalignant skeletal disorder characterized by the excessive proliferation of cellular fibrous connective tissue with woven bony trabeculae replacing the normal bone. It has been reported that the disease is caused by the mutations of guanine nucleotide-binding protein and alpha-stimulating (GNAS), encoding the alpha-subunit of Gs protein [1]. The mutation of GNAS 1 causes adenyl cyclase to be persistently active, and it produces increased cAMP activity which leads to hyperfunction of skeletal progenitor cells and abnormal osteoblasts. It has been reported that increasing interleukin-6 secretion, one of the downstream effectors of cAMP, may also take part in the process of FD by increasing the number of osteoclasts [2]. Meanwhile, constitutive activation of Gs stimulatory protein (Gas) leads to dysregulating proliferation of bone marrow stromal cells (BMSCs), which generates expansile lesions of fibrotic tissue and abnormal bone. Local bone remodeling regulation by BMSCs is also altered, and FD tissue is characterized by abundant osteoclast-like cells that may be essential for lesion expansion [3].

The disease is involved one (monostotic form) or more bones (polyostotic form) and furthermore it may be as part of the McCune-Albright syndrome (MAS), which is characterized by the following triad: fibrous dysplasia of bone, hyperfunctioning endocrinopathies, and/or cafe-au-lait spots. FD can be a troublesome disease, which may cause bone pain, bone deformity (especially shepherd’s crook deformity for the femur), or pathological fracture and usually begins in childhood. Many studies has reported that the femur and the maxillofacial region are the most affected sites [4].

The typical characteristic of varus Shepherd’s crook deformity in FD is associated with limb shortening, limping, and occasionally chronic fatigue fractures with disabling walk. Although aggressive orthopedic treatment is applied to the disease, recurrent fractures and relentless progression of the varus deformity are still characteristic involvements in FD. How to predict the progression of Shepherd’s crook deformity is still a challenging clinical problem.

Thus, the goal of the present study are to reveal the predictors of the progression of Shepherd’s crook deformity and to show the deformity of PFD being related to high speed of bone turnover, which is exactly reflected by the levels of serum ALP and calcium.

## Materials and methods

We presented a retrospective study analyzing 159 patients with FD with the postoperative pathological diagnosis, who were evaluated at our bone tumour center between January 2000 and September 2016. The diagnosis of FD was established based on a combination of clinical history, physical examination, radiographic findings, and histopathological diagnosis. Collection of information: the following diagnostic data were collected: baseline demographic features (sex, age at diagnosis, BMI), initial presenting symptoms, affected bone sites (monostotic FD or polyostotic FD), and histological results. Monitoring data were also recorded: skeletal events (fracture, surgery, malignancy) and radiological image during the follow-up. We had the approval that the clinical data of patients were used for this retrospective study from all patients during the follow-up and the Institutional Review Board of our hospital has approved our study.

The inclusion criteria for the present study were as follows: (1) with intact preoperative serum ALP, calcium, and phosphorus levels; (2) diagnosis as fibrous dysplasia by the confirmation of post-operative histology; and (3) with the intact clinical and follow-up data. The exclusion criteria were as follows: (1) patients lacking of record of preoperative serum ALP, calcium, and phosphorus levels and (2) incomplete medical records and loss of follow-up.

### Surgical technique and follow-up

For the lesion of limbs and axial skeletal, a cortical window was made and the lesion was resected. To fill the defect after intralesional curettage, autogenous bone graft or allograft bone or artificial bone or xenograft or cement or combination was used. Internal fixation was performed to restore the bone stability. The routine follow-up includes clinical examination and radiographs of the extremity every 3 months for the first 6 months, every 6 months for the first 3 years, and then annually.

### Statistical analysis

Statistical analyses were conducted using SPSS 19.0 (SPSS Inc., USA). We assessed differences between study groups using chi-square test for categorical variables. Continuous, normally distributed variables were analysed using a two-sample Student’s *t* test; continuous, non-normally distributed variables were analyzed using the Mann-Whitney test. A multivariate linear regression was applied to identify significant risk factors which had the close relationship with bone turnover markers. A logistic regression was applied to identify the risk factor of progression of shepherd’s crook deformity. A *P* value of < 0.05 was considered to indicate statistical significance.

## Results

### Clinical characteristics

The general clinical characteristics of the patients were summarized in Table 1. Eighty-five males and 74 females (male to female ratio 1.1:1) were recruited into the present study. The age of onset ranged from 5 to 75 years (average 28.6 ± 13.6 years). Thirty-two patients presented with PFD (20.1%) and 127 with MFD (79.9%). Local recurrences occurred in three (1.9%) out of 159 patients during the follow-up. No malignant transformation occurred in our cohort.

**Table 1:** Analysis of the risk factors for preoperative bone turnover markers for FD patients

	Pre-ALP	***P*** value	Pre-calcium	***P*** value	Pre-phosphorus	***P*** value
**Age**						
**≤ 14 (*N*** **= 23)**	359.8 ± 356.8	**<0.001**	2.45 ± 0.14	**<0.001**	1.75 ± 0.25	**<0.001**
**15–18 (*N*** **= 18)**	232.4 ± 178.3		2.42 ± 0.28		1.49 ± 0.23	
**≥ 19 (*N*** **= 118**)	90.4 ± 62.9		2.32 ± 0.13		1.23 ± 0.17	
**Gender**						
**Male (*N*** **= 85)**	189.68 ± 235.01	**0.001**	2.38 ± 0.18	**0.037**	1.32 ± 0.30	0.592
**Female (*N*** **= 74)**	94.65 ± 70.06		2.33 ± 0.13		1.35 ± 0.23	
**BMI**						
**< 25 (*N*** **= 117)**	156.26 ± 206.42	0.090	2.36 ± 0.17	0.183	1.36 ± 0.27	**0.043**
**≥ 25 (*N*** **= 42)**	115.36 ± 93.77		2.33 ± 0.14		1.26 ± 0.24	
**Location**						
**Limb (*N*** **= 122)**	159.24 ± 206.75	**0.004**	2.37 ± 0.17	**0.045**	1.36 ± 0.28	**0.008**
**Axial skeletal (*N*** **= 37)**	100.00 ± 47.97		2.31 ± 0.13		1.25 ± 0.19	
**Recurrence at presentation**						
**Yes (*N*** **= 12)**	150.17 ± 108.34	0.927	2.48 ± 0.12	**0.007**	1.31 ± 0.26	0.705
**No (*N*** **= 147)**	145.07 ± 189.18		2.34 ± 0.16		1.34 ± 0.27	
**Fracture**						
**Yes (*N*** **= 16)**	185.31 ± 153.56	0.363	2.36 ± 0.15	0.889	1.32 ± 0.37	0.819
**No (*N*** **= 143)**	140.99 ± 187.14		2.35 ± 0.16		1.34 ± 0.26	
**Type**						
**PFD (*N*** **= 32)**	238.13 ± 350.23	**0.001**	2.31 ± 0.15	0.121	1.24 ± 0.21	**0.008**
**MFD (*N*** **= 127)**	122.10 ± 97.93		2.36 ± 0.17		1.36 ± 0.28	
**Recurrence during follow-up**						
**Yes (*N*** **= 3)**	158.33 ± 87.59	0.903	2.31 ± 0.17	0.613	1.66 ± 0.30	**0.033**
**No (*N*** **= 156)**	145.21 ± 185.59		2.35 ± 0.16		1.33 ± 0.27	
**Deformity**						
**Yes (*N*** **= 17)**	313.12 ± 440.86	**<0.001**	2.26 ± 0.15	**0.009**	1.20 ± 0.27	**0.026**
**No (*N*** **= 142)**	125.15 ± 110.49		2.37 ± 0.16		1.35 ± 0.27	

### Influence of risk factors on the preoperative bone turnover markers

**Fig. 1:**
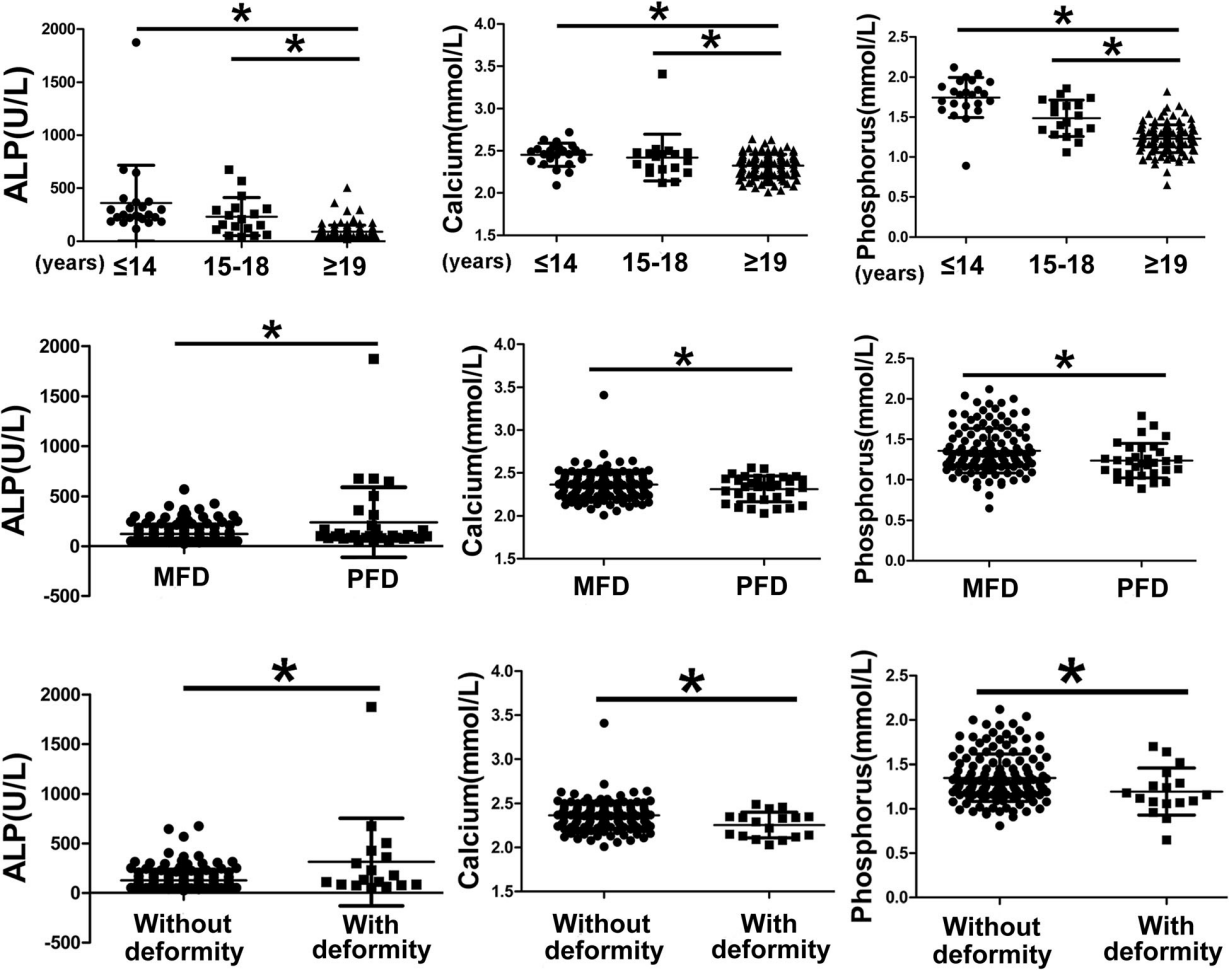
The relationships between risk factors and preoperative bone turnover markers

The univariate analysis of preoperative bone turnover markers was shown in Table 1. Age, gender, lesion location, lesion type, and shepherd’s crook deformity had a close relationship with preoperative ALP level in the univariate analysis. Children and teenager patients had significantly higher level of ALP than adults. The reference range of ALP level for children suggested by Stanley was shown in Table 2 [5]. For the evaluation of the rate of abnormal preoperative ALP for patients with FD, the statistical analysis showed patients with PFD and shepherd’s crook deformity were significant risk factors for the abnormal rate of ALP (Table 3). The multivariate analysis showed age, gender, lesion type, and shepherd’s crook deformity had the significant relationship with the preoperative serum ALP level. Meanwhile, it was the age and shepherd’s crook deformity for the preoperative serum calcium level (Fig. 1).

**Table 2:** The reference values of ALP for children and adults

Age	Value (U/L)	
**1–9 years**	145–420	
**10–11 years**	140–560	
	**Male**	**Female**
**12–13 years**	200–495	105–420
**14–15 years**	130–525	70–230
**16–19 years**	65–260	50–130
**> 19 years**	45–125	50–135

**Table 3:** Evaluation of the rate of abnormal preoperative ALP for patients with FD

	Abnormal no.	Abnormal rate	***P*** value (*χ*^**2**^)
**Age**			
**≤ 14 (*N*** **= 23)**	3	13.0%	0.481
**15–18 (*N*** **= 18)**	4	22.2%	
**≥ 19 (*N*** **= 118)**	14	11.9%	
**Gender**			
**Male (*N*** **= 85)**	16	18.8%	0.101
**Female (*N*** **= 74)**	6	8.1%	
**BMI**			
**< 25 (*N*** **= 120)**	17	14.2%	0.786
**≥ 25 (*N*** **= 39)**	4	10.3%	
**Location**			
**Limb (*N*** **= 122)**	15	12.3%	0.581
**Axial skeletal (*N*** **= 37)**	6	16.2%	
**Recurrence at presentation**			
**Yes (*N*** **= 12)**	2	16.7%	0.661
**No (*N*** **= 147)**	19	12.9%	
**Pathological fracture**			
**Yes (*N*** **= 16)**	4	25%	0.232
**No (*N*** **= 143)**	17	11.9%	
**Type**			
**PFD (*N*** **= 32)**	9	28.1%	**0.016**
**MFD (*N*** **= 127)**	12	9.4%	
**Recurrence during follow-up**			
**Yes (*N*** **= 3)**	0	0	1.000
**No (*N*** **= 156)**	21	13.5%	
**Deformity**			
**Yes (*N*** **= 17)**	7	41.2%	**0.002**
**No (*N*** **= 142)**	14	9.9%

### Effect of surgical procedure on the bone turnover markers

**Fig. 2:**
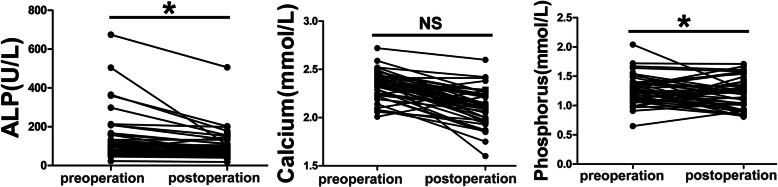
The comparison between the preoperative and postoperative bone turnover markers

Forty-six patients with intact postoperative chemical determinations were included for the analysis of influence of surgery on bone turnover markers for FD. The result revealed that there was a significant difference between the preoperative and postoperative ALP and calcium levels (*P* < 0.05) (Table 4). For FD patients, the surgical procedure could significantly reduce the ALP and calcium levels (Fig. 2).

**Table 4:** The change of bone metabolism after lesion resection for patients with FD (*N* = 46)

	ALP	Calcium	Phosphorus
**Preoperation**	129.98 ± 126.58	2.34 ± 0.14	1.25 ± 0.25
**Postoperation**	92.91 ± 75.81	2.11 ± 0.19	1.18 ± 0.25
***P*** **value**	**0.001**	**< 0.001**	0.099

### Analysis of predictors for shepherd’s crook deformity progression

Pathological fracture, FD type, and preoperative ALP had a significant correlationship with shepherd’s crook deformity in the univariate analysis (Table 5). Furthermore, the multivariate analysis showed FD type and preoperative ALP were the risk factors of shepherd’s crook deformity. The preoperative ALP level of FD patients with shepherd’s crook deformity was obviously higher than that without deformity, and the preoperative calcium and phosphorus levels of FD patients with deformity were significantly lower than that without deformity (*P* < 0.05, Table 6).

**Table 5:** Analysis of the risk factors of shepherd’s crook deformity for FD patients

	Deformity no.	Non-deformity no.	***P*** value (*χ*^**2**^)
**Age**			
**≤ 14 (*N*** **= 23)**	2	21	0.669
**15-18 (*N*** **= 18)**	3	15	
**≥ 19 (*N*** **= 118)**	12	106	
**Gender**			
**Male (*N*** **= 85)**	7	78	0.313
**Female (*N*** **= 74)**	10	64	
**BMI**			
**< 25 (*N*** **= 120)**	15	105	0.246
**≥ 25 (*N*** **= 39)**	2	37	
**Location**			
**Limb (*N*** **= 122)**	15	107	0.364
**Axial skeletal (*N*** **= 37)**	2	35	
**Recurrence at presentation**			
**Yes (*N*** **= 12)**	0	12	0.366
**No (*N*** **= 147)**	17	130	
**Pathological fracture**			
**Yes (*N*** **= 16)**	5	11	**0.016**
**No (*N*** **= 143)**	12	131	
**Type**			
**PFD (*N*** **= 32)**	10	22	**<0.001**
**MFD (*N*** **= 127)**	7	120	
**Preoperative ALP**			
**Normal (*N*** **= 138)**	10	128	**0.002**
**Abnormal (*N*** **= 21)**	7	14

**Table 6:** The bone turnover markers for FD patients with and without shepherd’s crook deformity

	ALP	Calcium	Phosphorus
**With deformity (*N*** **= 17)**	313.12 ± 440.86	2.26 ± 0.15	1.20 ± 0.27
**Without deformity (*N*** **= 142)**	125.15 ± 110.49	2.37 ± 0.16	1.35 ± 0.27
***P*** **value**	**< 0.001**	**0.008**	**0.024**

**Fig. 3:**
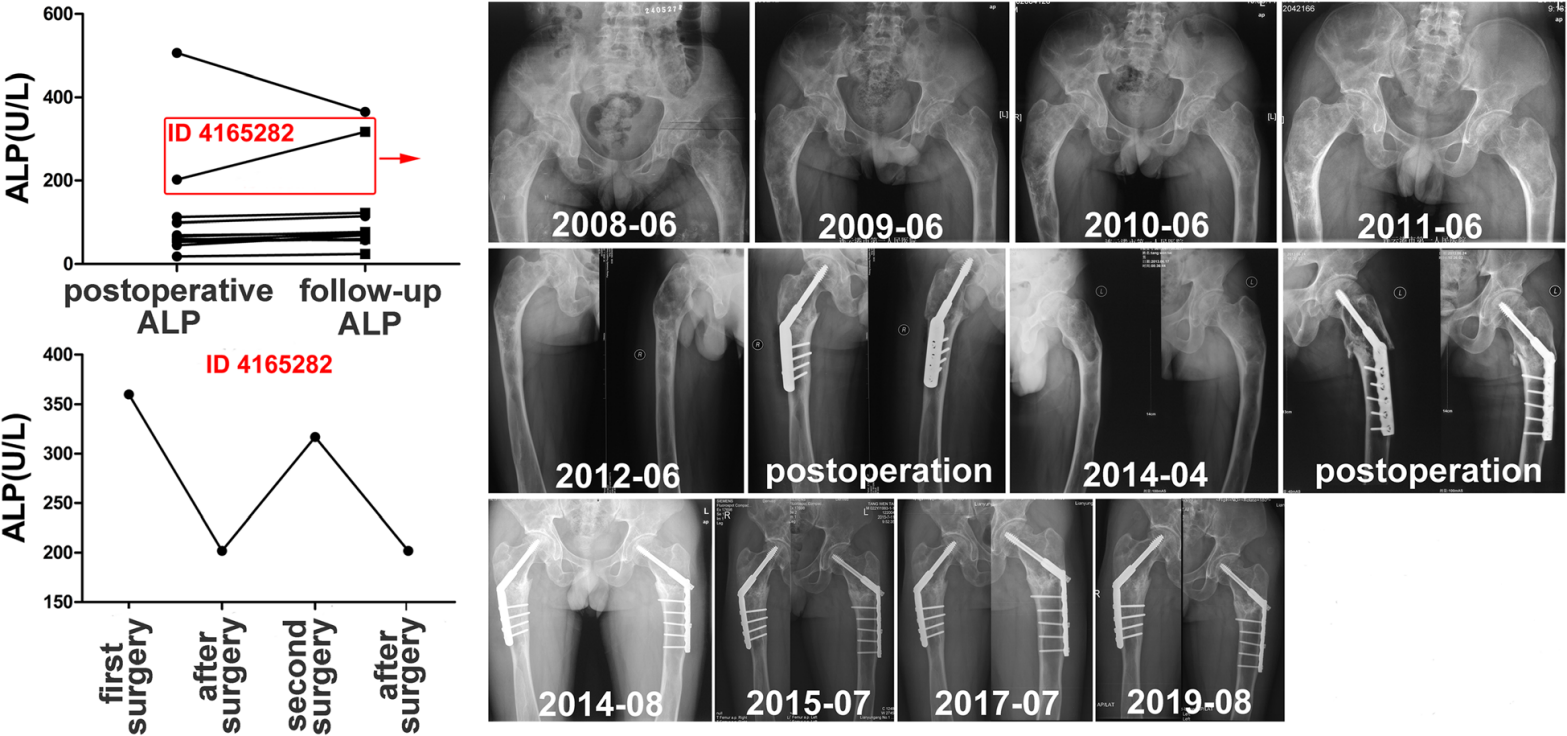
Eleven patients were recruited to assess the change of ALP level during the follow-up. A 19-year-old male patient with bilateral shepherd’s crook deformity in the femurs. The change of ALP was correlated to the progression of shepherd’s crook deformity

Eleven patients were recruited to assess the change of ALP level during the follow-up. A 19-year-old male patient had bilateral shepherd’s crook deformity in the femurs. He received the first intralesional curettage and corrective surgery for shepherd’s crook deformity in the femur. The postoperative ALP (360 U/L) was significantly decreasing to 202 U/L. However, the ALP level increased to the high level (317 U/L) during the follow-up and at that time X-ray showed shepherd’s crook deformity severely progressing. He received the second surgical procedure for another femur (Fig. 3).

## Discussion

Fibrous dysplasia (FD) is a rare bone disorder in which normal intramedullary bone is replaced by fibro-osseous tissue. It has been reported that the disease is associated to mutations of guanine nucleotide-binding protein and alpha-stimulating and this gene encodes the alpha subunit of Gs protein. The mutation of GNAS 1 causes adenyl cyclase to be persistently active, and it produces increased cAMP activity which leads to hyperfunction of skeletal progenitor cells and abnormal osteoblasts [1]. To date, it is controversial which factors are to be closely associated with the prognosis of FD patients. Some opinions are that the deformity obviously influences the prognosis of either the adult or children and the progressive varus shepherd’s crook deformity in fibrous dysplasia is associated with limb shortening, limping, and occasionally chronic fatigue fractures with disabling walk [6]. Benhamou et al. reported that the polyostotic form was the main risk factor of a clinical poorer outcome [7]. Thus, it is more crucial to detect a prognostic marker for FD.

Alkaline phosphatase (ALP) is an important hydrolase enzyme related to osteoblastic activity and is used as a prognostic marker for bone-related malignant tumors especially for osteosarcoma. ALP can be used as a promising prognostic marker for malignant tumors. However, the significance of serum ALP level for patients with bone fibrous dysplasia is unclear and there are more controversies on the prognostic effect. Park et al. reported the relationship between local recurrence and postoperative serum ALP level. They observed an abrupt increase in ALP level before the recurrence of craniofacial fibrous dysplasia in seven patients. These seven patients with regrowth had abnormally high postoperative ALP level at a time point during the follow-up. The time point of the abrupt rise in ALP was correlated with the regrowth of FD, confirmed by CT scanning. Their result recommended the postoperative serum ALP level as a reliable marker for predicting the progress of CFD [8]. Hussein et al. also revealed that serum ALP was significantly associated with tumour recurrence during long-term follow-up [9]. However, Ma et al. showed patients with recurrence did not show significantly higher levels and abnormal rates of ALP than those without recurrence. Both the mean level and the mean abnormal rate of ALP in patients with recurrence were lower than in patients without recurrence. They think that the elevated serum ALP levels observed in our patients preoperatively may be due to the elevated levels of calcitonin, because calcitonin increases intracellular ALP through the action of cAMP. Also, the age relationship might be due to normal physiological bone metabolism rather than the tumor itself. Thus, they concluded that preoperative ALP was an unsuitable prognostic marker for postoperative tumor behavior [10]. Our present study showed that the preoperative serum ALP and calcium levels can not predict the local recurrence but the progression of shepherd’s crook deformity. Thus, we can screen the deformity progression in the bone by monitoring the serum ALP and calcium levels. Although no malignant transformation of FD occurred in our cohort, the malignant change into osteosarcoma was reported in the previous study. Thus, it needs to be considered to make a close follow-up necessary [11–14].

Adolescents have a higher level of ALP than adults because of rapid growth and boys have a higher ALP level than girls. These differences must be taken into the consideration when evaluating ALP level in patients of different ages and genders. Thus, the reference range of ALP level for children suggested by Stanley is used in our study [5]. Our results showed that the level of preoperative ALP was closely related to the lesion type and shepherd’s crook deformity. The PFD patients showed both significantly higher level and abnormal rate of ALP compared to MFD patients. Thus, it demonstrated that the level of ALP correlated with the extent of disease activity. These results were partially in agreement with Ma’s study [10]. We also found that preoperative ALP level was closely related to age and FD type (monostotic or polyostotic), which was consistent with the conclusion of the previous study in the literature [10]. However, some authors supported the opposite conclusion that ALP level did not have a close relationship with the age and FD type [9]. Our results showed that there was no significant difference in the preoperative ALP level in patients with or without local recurrence. Due to the limited number of recurrent patients (three cases), whether ALP level was related to the local recurrence was unclear.

One of the most common clinical issues in FD is pathological fracture. The risk factor of fracture for FD patient is unclear. Leet et al. reported that patients with metabolic abnormalities sustained their first fracture at an earlier age and had a higher lifetime rate, compared to patients with PFD alone. The risk of pathological fracture was aggravated by metabolic derangements, such as concomitant metabolic dysfunction (MAS) with hyperfunctioning endocrinopathy and/or phosphaturia. However, the influence of metabolic derangements on the pathological fracture of FD patient was not evaluated due to lack of endocrinic information [15]. Ippolito et al. reported that 61% of the femoral deformity presented at diagnosis switched to a more severe type of deformity within 3 years from diagnosis, whereas 87% either shifted to a more severe type of deformity or receiving the osteotomy [16]. In the present study, there was no statistical difference between the occurrence of pathological fracture and preoperative ALP level. Thus, in our experience, more attention should be paid to the severe shepherd’s crook deformity and it can increase the pathological fracture.

Combination of the osteotomy should be considered for the shepherd’s crook deformity. The pathological fracture combined with the severe deformity in the femur is more common for FD patients. The osteotomy combination with the plate or intramedullary nail could provide the valgus realignment for the shepherd’s crook deformity. In some special cases, the pathological fracture occurs in the proximal femur with shepherd’s crook deformity. This condition is more complicated and more attention should be paid. In the literature, Al-Mouazzen et al. have reported a case with the shepherd’s crook deformity secondary to FD, who suffered from an intra-capsular femoral neck fracture. The closed reduction and internal fixation with cannulated screws were initially performed. Unfortunately, a guide wire broke inside the femoral head during the procedure. And then, the patient received a second operation to remove the screws and correct the varus deformity using a closing-wedge femoral osteotomy and the function was recovered to the normal level at 1-year follow-up [17]. For the repression of shepherd’s crook deformity development, medical treatment such as the use of bisphosphonates also has been shown to be effective in the relieving pain and decreasing bone resorption [18]. Boyce et al. showed that treatment FD with denosumab, whose immunohistochemical staining on a pretreated bone biopsy specimen revealed marked RANKL expression, led to the dramatic reduction of bone destroy expansion and FD-related bone pain [19]. These results revealed that improving the unbalanced bone turnover could increase the bone density and reduce the bony lesion. In our cohort, the use of bisphosphonates is recommended for FD patients with the severe shepherd’s crook deformity.

This study had several limitations. Firstly, it should be noted that the number of cases was still limited and could not accurately analyze the relationship between the local recurrence and bone turnover markers. Secondly, the other bone turnover markers were not evaluated and their relationships were in the process of detection.

In conclusion, PFD with a higher serum ALP level has an obvious tendency to progress severely and risk factors of progression to the deformity are the condition of bony metabolism and FD type. The deformity of PFD may be related to high speed of bone turnover, which is exactly reflected by levels of serum ALP and calcium. Evaluation of patients with FD should include a thorough evaluation of calcium/phosphate metabolism and bone turnover, except the proper pathological and radiographic assessment. If possible, endocrine profile is also needed to evaluate.

## Data Availability

The data and materials are available from the medical records of the department of Peking University People’s Hospital. The datasets used and analyzed during the current study are available from the corresponding author on reasonable request.

## Abbreviations

FD: Fibrous dysplasia
ALP: Alkaline phosphatase

## References

[1] Weinstein LS, Shenker A, Gejman PV, Merino MJ, Friedman E, Spiegel AM (1991). Activating mutations of the stimulatory G protein in the McCune-Albright syndrome. N Engl J Med..

[2] Yamamoto T, Ozono K, Kasayama S, Yoh K, Hiroshima K, Takagi M, Matsumoto S, Michigami T, Yamaoka K, Kishimoto T, Okada S (1996). Increased IL-6 production by cells isolated from the fibrous bone dysplasia tissues in patients with McCune-Albright Syndrome. J Clin Invest..

[3] de Castro LF, Burke AB, Wang HD, Tsai J, Florenzano P, Pan KS, Bhattacharyya N, Boyce AM, Gafni RI, Molinolo AA, Robey PG, Collins MT (2019). Activation of RANK/RANKL/OPG pathway is involved in the pathophysiology of fibrous dysplasia and associated with disease burden. J Bone Miner Res.

[4] Cheng J, Wang Y, Yu H, Wang D, Ye J, Jiang H, Wu Y, Shen G (2012). An epidemiological and clinical analysis of craniomaxillofacial fibrous dysplasia in a Chinese population. Orphanet J Rare Dis.

[5] Stanley F, Kliegman RM, Behrman RE, Stanton BF, St. Geme J, Schor N (2010). Reference intervals for laboratory tests and procedures. Nelson textbook of pediatrics.

[6] Paul SM, Gabor LR, Rudzinski S, Giovanni D, Boyce AM, Kelly MR, Collins MT (2014). Disease severity and functional factors associated with walking performance in polyostotic fibrous dysplasia. Bone.

[7] Benedetti Valentini M, Ippolito E, Catellani F, Farsetti P (2015). Internal fixation after fracture or osteotomy of the femur in young children with polyostotic fibrous dysplasia. J Pediatr Orthop.

[8] Park BY, Cheon YW, Kim YO, Pae NS, Lee WJ (2010). Prognosis for craniofacial fibrous dysplasia after incomplete resection: age and serum alkaline phosphatase. Int J Oral Maxillofac Surg.

[9] Hussein MA, Yun IS, Kim BO, Kim YO (2017). Craniofacial fibrous dysplasia: retrospective study on the relationship between the tumor volume changes and the circulating serum calcitonin and serum alkaline phosphatase. Ann Plast Surg.

[10] Ma J, Liang L, Gu B, Zhang H, Wen W, Liu H (2013). A retrospective study on craniofacial fibrous dysplasia: preoperative serum alkaline phosphatase as a prognostic marker?. J Craniomaxillofac Surg.

[11] Sugiura Y, Kanda H, Motoi N, Nomura K, Inamura K, Okada E, Matsumoto H, Shimoji T, Matsumoto S, Nakayama J, Takazawa Y, Ishikawa Y, Machinami R (2018). Osteosarcoma arising in fibrous dysplasia, confirmed by mutational analysis of GNAS gene. Pathol Res Pract.

[12] Qu N, Yao W, Cui X, Zhang H (2015). Malignant transformation in monostotic fibrous dysplasia: clinical features, imaging features, outcomes in 10 patients, and review. Medicine (Baltimore).

[13] Pack SE, Al Share AA, Quereshy FA, Baur DA (2016). Osteosarcoma of the mandible arising in fibrous dysplasia-a case report. J Craniomaxillofac Surg.

[14] Kammerer PW, Shabazfar N, Vorkhshori Makoie N, Moergel M, Al-Nawas B (2012). Clinical, therapeutic and prognostic features of osteosarcoma of the jaws—experience of 36 cases. J Craniomaxillofac Surg..

[15] Leet AI, Collins MT (2007). Current approach to fibrous dysplasia of bone and McCune-Albright syndrome. J Child Orthop..

[16] Ippolito E, Valentini MB, Lala R, De MF, Sorge R, Farsetti P (2016). Changing pattern of femoral deformity during growth in polyostotic fibrous dysplasia of the bone—an analysis of 46 cases. J Pediatr Orthop.

[17] Al-Mouazzen L, Rajakulendran K, Ahad N (2013). Fibrous dysplasia, shepherd’s crook deformity and an intra-capsular femoral neck fracture. Strategies Trauma Limb Reconstr.

[18] Wu D, Ma J, Bao S, Guan H (2015). Continuous effect with long-term safety in zoledronic acid therapy for polyostotic fibrous dysplasia with severe bone destruction. Rheumatol Int..

[19] Boyce AM, Chong WH, Yao J, Gafni RI, Kelly MH, Chamberlain CE, Bassim C, Cherman N, Ellsworth M, Kasa-Vubu JZ, Farley FA, Molinolo AA, Bhattacharyya N, Collins MT (2012). Denosumab treatment for fibrous dysplasia. J Bone Miner Res..

